# Dentifrices and Mouth-Washes

**Published:** 1908-03-15

**Authors:** Geo. W. Cook


### DENTIFRICES AND MOUTH-WASHES.

BY GEO. W. COOK, B.S., D.D.S.

Read before the Detroit Dental Society, December 12, 1907.

Dentifrice, in the last analysis of the word, may mean either a tooth powder or a tooth wash. The above title is given in about the way the profession of dentistry uses the two terms. A tooth powder, as we at the present time understand it, we always call a dentifrice. We apply the term mouth-wash to a liquid remedy that is capable of removing any foreign substance from the oral cavity. Its use has become quite frequent since the discovery of the pathogenic properties of bacteria, and since it has been discovered that a large number of the so-called parasitic forms of life are habitual inhabitants of the oral cavity.

Tooth powders, in their original application, were used as a means of removing, or at least keeping, substances from forming on the surfaces of the teeth, lest they remain there and mar the appearance of these organs. Tooth powders, according to history, as agents for cleaning and brightening the teeth, came into use many centuries ago; and various compounds were prepared by certain races and classes of people for enhancing the beauty of their teeth. Many of the uncivilized races resorted to this means of brightening the surfaces of their teeth to make themselves attractive. From the very earliest data in medical antiquity we find that tooth powders, or something that served that purpose, were used; but at that time, as we have stated, they were used as agents to polish the teeth. Common wood ash and various agents of like character were used to rub on the teeth with sticks; and we might say that the modern tooth-brush is the outcome of an attempt many centuries ago to prepare an appliance with which a powder or gritty substance might be applied to the teeth. At that time the use of the powder and brush was confined very largely to the anterior teeth and was only for the purpose of making them ornamental, the users being absolutely unmindful of the deleterious effect they would have upon their teeth. From a study of the subject before hygiene was known, tooth powders seem to have been used with equally as much intelligence as at the present time.

Modern dentistry has brought great revolutionary ideas into operation and in some respects has rendered great service to the race; but with it has come some most deleterious influences for destroying the beauty and long usefulness of the natural dentures. In our modern time, and with modern ideas of beauty and hygiene, has continued the enthusiasm for rendering the teeth one of the ornamental characteristics of the face. Wherever there is a desire on the part of the public in general to go to extremes there comes to the front the commercial instincts of the race, to prepare and sell to the public commercial and secret remedies whether they are a class of commodities that are useful to the people or not. In many respects these remedies are not only useless to the individuals, but sometimes a great detriment, especially if put into the hands of these enthusiastic persons who wish to make their teeth not only useful but ornamental and attractive.

A tooth powder, which consists of a chalky substance, should be used only for the purpose of removing the inorganic and mucilaginous substances that attach to the surfaces of the teeth. This is a useful and very essential process, but it must be borne in mind that the extremes now practiced will do great harm, for we all are aware how unintelligent even the dentists themselves are in using tooth powders and other agents for the purpose of cleansing the teeth.

The classic work of the late Dr. Miller on the effects of tooth powder and the brush on the surfaces of the teeth, is so recently before our minds, that I shall only allude to the fact that he demonstrated how easily and how rapidly a tooth might be brushed away by the use of a brush and tooth powder. I need only state that these agents are the greatest possible assistants in the causation of erosion of the teeth. I do not wish to be misunderstood as saying that tooth powders and tooth brushes are the sole cause of erosion, but I do wish to emphasize the fact that they are important factors in this process.

The object of a tooth powder is principally to cleanse the teeth, or in other words to remove all substances that remain about or between the teeth. Now, as a profession we have left this important branch of our therapeutics to commercial individuals, and it is a well established fact, that wherever commercialism enters the field of dentistry, science must give way. Especially is this true when it comes to medicinal agents; for scientific therapeutics has never come to the dental profession in a way that has materially benefited it.

We have not had an agent in a great many years that is any improvement over the early attempt at getting up a good common-sense tooth powder. A tooth powder given to the profession about thirty-five years ago was just as good as anything since that time; and, in fact, there are a number of tooth powders that have agents in them that are absolutely deleterious both to the tooth substance and to the mucous membrane. The mixed saliva contains mucous cells with more or less mucin or colloidial substance with which a tooth powder will become more or less a pasty mass, which may contain a large number of bacteria. These micro-organisms will soon grow, and there usually is present enough organic material in the mass of glue-like substance to carry on the growth of bacteria. The statement will probably be made that we should incorporate antiseptics with all our tooth powders. Any such substance will filter out of a powder in the mouth in from three to eleven minutes so that not a trace of it can be found. With these facts at our hand, then, the usefulness of a tooth powder can only serve one purpose, and that is to keep the surface of the teeth clean and free from extraneous substances. Now, with the knowledge that these agents will in certain mouths cut away the tooth tissue with great rapidity, it leaves us in doubt as to whether or not we should use them at all.

If a tooth powder is to be used, it should not be used until the dentist has determined just what it contains, especially as to the fineness of the grit. Any tooth powder containing cuttle-fish or pumice should be excluded, as these agents will cut away tooth substance almost as fast as a sand-paper disk. A tooth powder containing 1½ percent soap will cause, when used twice a day for several weeks, a degeneration of the mucous tissue. The soap from soap bark will cause this last named condition more rapidly than the ordinary castile soap.

The soft tissue cell that is of the most importance to the teeth in the oral cavity is the unicellular glandular epithelium of the mucous membrane. If the preservation of this tissue is not maintained, then our function in giving the best care to this special field is a failure. Our first function is to treat the teeth without any way interfering with the surrounding tissue; and, when our treatment is of such a nature as to render the function of the mucous tissue about the teeth degenerate, then our treatment is not in accordance with the most scientific phenomenon known to biologists. If we look into the pharmacology of most agents that are used in the oral cavity as mouth-washes, we will find that they will, if accompanied with friction, cause degeneration of the mucous tissue. It is quite essential then that the condition for which a tooth powder or mouth-wash is prescribed be given some consideration.

There are incorporated in tooth powders agents that are usually spoken of as drugs. The word drug means a crude remedy used in medicine. Practicing medicine is the art or science of healing disease, especially by the employment of a remedy in the treatment of a disease. With this understanding, a tooth powder would naturally come under the head of preventive medicines. Now it is a question whether or not this branch of medicine should be prescribed by specially educated individuals whose trainings have been such as to make them competent to differentiate as to whether an astringent or a stimulant shall be used in a certain case. If the observer is sufficiently skilled, he may determine whether or not a preventive remedy is required. Some questions naturally arise: Shall preventive remedies be prescribed, and under what condition shall they be indicated in mouths in which there are present sound teeth and mucous tissue, and where there are no irregularities, in other words, where there is a normal oral cavity? I should say, by no means need we prescribe antiseptic tooth powders and mouth washes.

If teeth are not in their normal relations to each other the mucous tissue is not in a normal condition. Then it is the duty of the clinician to study the individual abnormalities and apply such remedies as the case may demand. If it is studied from a physiological and pharmacological standpoint it will be possible to reach a base of scientific and practical therapeutics. It should be borne in mind that when a tooth powder or mouth-wash is prescribed when there is no specific pathological lesion present, it comes under the head of preventive therapeutics. In prescribing a preventive remedy it should be prescribed as a specific against the possible occurrence of a specific disease process.

If we accept the biological causation of disease, we must then agree that a remedy that will prevent caries of teeth will not always prevent diseases that manifest themselves in the mucous tissue, and vice versa. However, it is universally attested that the original use of these remedies was for the preventing of carious conditions of the teeth alone; and, in the effort to prevent this pathological condition of the teeth, almost every day there are remedies prescribed that may prevent this disease process. But in establishing immunity or preventing decay, the remedy may be of such a nature as to cause pathological changes in the soft tissues of the parts about the teeth. Any of these agents may not make any appreciative change in the mucous epithelial structure for a considerable time, but the gradual degeneration may at once burst into a flame of pathological changes that may pass into a chronic pathological state and remain that way until the teeth are completely lost. When these remedies are causing such a condition of epithelial degeneration, they should be withdrawn at once. The misfortune is that these pathological processes are seldom recognized until the process has reached a chronic state. When that state of pathology has been reached, it is difficult to know whether the remedy or the bacteria is causing the disease process.

There are many agents that are being daily used and recommended by the dental profession, which, in my hands and attested by various processes of applying them to the epithelial mucous tissue, have proven to be most essential in bringing about certain tissue changes that are pathological in their general characteristics. These agents have under certain circumstances proved to be specific in the causation of erosion and may indirectly have caused pyorrhea alveolaris. I have taken large quantities of tooth powders and mouth-washes and applied them to culture medias in various ways. As a result bacteria grew and fermentation increased one-half of one percent in acidity over the controls that had none of the remedies in them. The length of time that it takes the most efficient disinfectant to act upon the bacterial cell, though even that agent could be applied to the mucous membrane, is so great that one may hope to remove the bacteria from the oral mucous membrane only in a mechanical way, in other words, in washing them out of the mouth. Experiments also proved that, when these most potent remedies were used in the oral cavity, growth did not take place in the culture media as rapidly as they did in the control. We demonstrated, however, that by making a chemical test for the agents used as disinfectants in the mouth, traces could be found, in the culture medias showing that they acted rather upon the bacteria after they had been transferred to the artificial media than while they were in the mouth.

With such facts as these before us, we are inclined to repeat a statement formerly made upon this question—that, at the present time there is not a remedy on the market that is capable of acting as a disinfectant to the oral mucous membrane except by mechanical action only. Besides, the agents that we look upon as being the most potent in the destruction or prevention of the growth of bacteria, will produce more deleterious effects upon the mucous epithelial structure than they will upon bacterial life. I have proved by the application, on a particular tissue, of certain agents like thymol, trikresol, formalin in the most diluted solutions, bichloride of mercury 1-1000, boracic acid, and various of the hydro carbon compounds, by moistening a pledget of cotton and placing it on a particular location and holding it there from one to two minutes and a half three times a day, that each is the cause of certain pathological changes in that particular spot, the phenomenon of which did not appear in the tissue in other parts of the mucous epithelium. These pathological changes differed as much in their microscopic appearance as each individual differed from another. I remember one case in particular where forty-two treatments twice a day, with a two percent solution of thymol applied along the gingival border or just above the gingiva over a cuspid tooth, produced degeneration of the epithelial structure, into the characteristic mucoid degeneration of tissue that is well described in the literature on degeneration. This same solution was applied in the same location in the mouth of another individual in which there appeared a hypertrophied growth of the epithelial structure; the result differed so characteristically from the other that you would think that the same agent could not possibly have produced the two pathological changes. So it is a wonderfully complex task to say just what we can or can not use as a dentifrice.

It only requires a brief consideration to see the adaptive capacity of the tissues of the oral cavity. It would consume so much time even to touch upon this phase of the biological phenomena, so constantly before us in the study of any condition of the oral cavity, that we cannot discuss this phase of the subject. But wonderful as it may seem, my treating and studying the tissues in the oral cavity and especially around the teeth, has made me wonder if really we know anything about the physiology, to say nothing of the pathology that comes under our observation. In perfectly normal persons, or we might say those who measure up to the standard of what we call physiological process, about 20% transfer to the saliva from the tissues and fluids of the body, immunizing substances. The other 80% of these people, so-called normal, have varying degrees of immunity or bacteriolytic action; while a large percent of the tired and nervous people, who do not have the standard normal conditions, are constantly susceptible to one or the other agent as applied to the mucous tissue, and also have a restraining effect upon one class of bacteria, while it practically increases the virulent properties of the others. When I say virulent properties, I mean those properties which are the physiological type of bacteria.

So it will be seen that in the study of remedies and in applying them in the oral cavity, we cannot lay down any specific rule whereby we can prescribe remedies as dentifrices, or can recommend their universal use, any more than we can recommend a particular treatment as truly specific in the immunizing or cure of any disease. But I believe that if an individual can be made by us to throw off more water with the salivary and mucous products, so that it will become a more universal solvent of the mucoid and colloidial substance, that we will render a greater service to our patients than all the therapeutists could prescribe in a hundred years.

I should like to make two or three suggestions here. First, do not prescribe astringent solutions that will act in a very appreciable manner on the mucous epithelium. Prescribe only stimulants that will act in the very mildest manner, for these two agents are the ones that will cause degenerative changes in the mucous tissue. If you have decided that you must prescribe any of these things, unless they are for specific conditions that exist as pathological lesions, do so in the mildest manner possible, because I can assure you that their action on the mucous membrane is of greater detriment than the benefits would be. When there is some specific condition that you prescribe a certain drug for, you should know exactly what pathological changes are taking place in the tissues.

Now, as for tooth powders and tooth pastes, all the difference there is between the two is the addition of a sufficient amount of glycerine to make a thin pasty mass of the powder. I will here append a prescription that was suggested by Dr. C. E. Jones of Chicago, after he had gone over a large number of formulae, and after he had made up hundreds of batches of tooth powders that are more or less known to the profession. This mass as a powder should never be used except when it has been filtered through two thicknesses of ordinary silk. A powder that will not pass through two thicknesses of silk will cut away tooth substance in the mouths of some people so rapidly that the patient and dentist will become alarmed at the wonderful development of erosion.

There are times when even an apparently healthy tissue would be benefited by some agent that would stimulate the physical properties of these cellular elements. But the point I wish to make is that we must prescribe certain agents with due consideration as to what effect they will have on healthy tissue cells. The formula that I have given here, prepared in the proper manner, is the only tooth powder out of forty-two different samples tested in the laboratory in the last five years that will not produce either rapid degeneration of the tissue cells or produce certain eroded conditions in the mouth of animals as well as in man. The real efficiency of a tooth powder lies principally in its having no effects on the tissues except in a mechanical way.

It will be impossible for me to go into a discussion of mouth-washes, because to my mind this involves one of the most interesting problems in dental therapeutics. Suffice it to say that a mouth-wash should be prescribed, not with a view of rendering the oral mucous membrane free from bacterial life through its action on the bacteria themselves, but that it can possibly operate only as a mechanical agent. If it is only to be used occasionally it can be made stronger. The main plea that I make in this paper is for the preservation of gum tissue, and we cannot preserve this important tissue structure if we use all sorts and kinds of agents, and especially those unknown to us. The time is past for the profession of dentistry to rely upon empirical remedies and practice empirical therapeutics.

ANTACID TOOTH POWDER.

| | | | |
|---|---|---|---|
| R | Magnesii Oxidi | 5. | Forms milk of magnesia with water. |
| | Calcii carbonatis | 10. | Precipitated chalk, grit. |
| | Magnesii peroxidi | 2. | Oxidizing agent, O is liberated when brought in contact with lactic acid. |
| | Menthol | 0.20 | Antiseptic, cooling agent. |
| | Benzosulphinidi | 0.10 | Flavor, (saccharinum). |
| | Olei Menthae pip | 0.20 | Flavor aromatic. |
| | M. | | |

Sig. Apply with brush.

Mix first three ingredients together in mortar. Dissolve the benzosulphinidum and menthol in the oil and while adding to the powder, triturate thoroughly.

DISCUSSION.

DR. T. J. COLLINS: I like this paper of Dr. Cook's because it approaches the subject, and its proper solution in the only way that it can be solved, along the line of clinical observation, experience and scientific research. In many of our minds, enthusiasm seems to take the place of science, and we are too ready to bow to idols of wood and stone, and to cling to dogmatic assertion. Many absurd claims are made as to the various properties of many of the proprietary tooth powders and mouth-washes that are on the market to-day; a great many of the statements are absolutely contradictory.

It seems to me quite plain that the tooth powder and mouth wash business should not be left entirely in the hands of commercial men. I do not mean to say but what some of these concerns do make some effort to prepare a scientific powder and mouth-wash, but, so far as I can learn, most of their investigations have been along the lines of preparing a germicidal or antiseptic preparation with no reference whatever to the tissues. Now it seems clear to me that the preparation of mouth-washes and tooth powders should be left in the hands of some one who will take professional interest in the matter. We cannot, however, blame the commercial men as they only want to do business. I am pleased to have Dr. Cook with us tonight and have listened with profit to his address. I trust he will go on with his researches to some definite conclusion. We want more men, if possible, to work along these lines.

Dr. N. S. Hoff: This is a most interesting paper, and it is a most interesting subject; a subject in which we all ought to be interested, and of which we ought to have more definite knowledge. The essayist's statements in the paper are excellent, particularly in line with the experience we have had in the use of tooth powders and mouth-washes.

We have a great many tooth powders, and all of them are quoted by the commercial authorities as being "curative," having antiseptic or germicidal values. The essayist makes the statement that in most dentifrices the antiseptics are so out of proportion to the other ingredients, and in the powders that they soon become dissolved and so diluted by the saliva in the mouth that after a few minutes of time they have no antiseptic properties whatever. Now if they have no greater range of value than that, their power is so insignificant that they would have no practical use. I do not see how the essayist harmonizes this statement with his other statement that these same medicinal reagents in the mouth create such havoc on the mucous cells. I am prepared to believe that a great many of the reagents used in the powders do harm and mischief. I believe there is no tooth powder that has ever been put upon the market that is calculated to do so much injury to the mucous membrane as the recently exploited peroxide of hydrogen powders. From what little experience I have had with them, I have found that they are prone to produce decided irritation of the mucous membrane in the mouth, causing gingivitis, and stomatitis in some mouths, and I predict that all this class of powders will be laid aside before long. Large percentages of soap or alkalies are exceedingly detrimental, producing congestion of the mucous membrane, irritation of the gingiva and absorption of the gum around the teeth and sensitiveness of the exposed pericementum. Soaps in the tooth powders are useful detergents but liable to be misused and produce bad results. I also think that irritant aromatics, used to a considerable extent, are injurious. We have on the market a number of tooth pastes—one made in this city, a very excellent product of its kind, but I have abandoned its use among my patients, or have advised its discontinuance altogether, because the strong aromatic substance which it contains over stimulate the gum tissues. Incidentally I would also say that it has other detrimental ingredients that might just as well be left out. All essential oils contain insoluble materials, which deposit on the teeth and produce a stain on the necks of the teeth that is very difficult to remove. Therefore because of this strong aromatic reaction and the staining properties they are not desirable dentifrices to use. I feel that the essayist has really given us the key to the whole matter when he says that we must prescribe our own powders. Just how we are going to do that is a serious problem, but we ought to prescribe our own dentifrice in every case. There is no single tooth powder that anyone can devise that will be useful in every or any large number of cases, or for all or any considerable length of time. The dentifrice should be changed from time to time as conditions in the mouth change. Necessarily we should see our patients more frequently in order to note the conditions that are developing in the mouth, so that we may change the dentifrice or the manner of using it, or advise its discontinuance altogether when advisable.

As a rule, most of our patients do not use a sufficient amount of tooth powder, but some do. Some are very conscientious about this matter and use tht powder a little too religiously with the tooth-brush. I firmly believe in tooth powder ,but I also firmly believe that it should be a simple powder containing less injurious medicinal reagents than most of the powders that we have now available. The simpler the powder the better, providing it is effective. I do not think precipitated chalk alone is sufficient, as many do; it is an excellent basis but it should be combined with some more abrasing reagent, such as magnesium carbonate or small quantities of fine powdered cutt e-fish or powdered pumice. Carbonate of magnesia combined with chalk, and, if you wish, something to make it sweet to induce patients to use it more freely, a little saccharin will sweeten and add disinfectant value.

Our whole object in using the powder is to keep the teeth clean, as the essayist has said, and where we go further than that and attempt to cure diseases of the mouth with a dentifrice, it seems to me we are asking too much. I have never seen actual diseases of the mouth corrected by tooth powders or mouth-washes. I have seen decay retarded by keeping the teeth and mouth clean. We cannot control our patients if we prescribe a proprietary tooth powder, they will buy without consulting us and use the powders excessively, and longer than we intended they should, and long after the purpose for which that powder was prescribed has been effected, they may use it until it acts detrimentally. If we want to get the best results and act professionally it seems to me that we should compound our dentifrices ourselves, or have them compounded by a competent druggist on our order. The patients will then get powder as they need it, and if we find that they have used it as long as desirable we should then change it and give them something more simple or more radical as the case may require. We cannot recommend every nostrum that is put upon the market, because our patients get the habit and would use the dentifrice year after year without further advice, which might be to their detriment. I have about come to the conclusion that we should not prescribe any of the miscellaneous commercial powders. Patients come to our offices and ask what tooth powder we recommend, and because we have no formula in mind or don't want to take the time or trouble to examine the patients and prescribe for him properly, we recommend some powder on the market. The whole question, it seems to me, is one that we want to study more carefully than we have, and get out of the habit of prescribing commercial tooth powders, just because it is easiest, and prescribe those powders that we know the patient should have, and meanwhile keep our patients under observation so that we may know when to change the dentifrice to something that will meet the varying requirements.

Dr. A. L. LeGro: I must confess that this is a subject to which I have given very little attention, but it seems to me that the natural deduction of this paper tonight is that we should confine ourselves to very simple tooth powders until, as Dr. Cook says, scientific therapeutics come to our aid. Now I have been wandering blindly along in this tooth-powder business for a number of years; in fact, I have had the simplest formula that I could possibly concoct, put up for my patients. I never have gone into the commercial side of it at all, and I have given this to my patients never thinking that any one of the ingredients that enter into the make-up of this powder would deteriorate or disintegrate the epithelial structure. This is new to me and I have never given it the least bit of study. I had a talk with Dr. Jones of Chicago upon this same subject, and I think very much as he does, that when you can take any tooth powder on the market and introduce into it a culture of the germs from the mouth, and find these germs will propagate readily, that such a tooth powder practically is a culture medium. And when that is true of almost every powder on the market, it is high time for us to start at the simplest scientific basis and build up again. Why can't our scientific therapeutists come to our aid? It is unfortunate that we have not more men in the profession indulging in this original research work. Such men as Dr. Cook, I know, have made a great sacrifice, both in time and finances, to arrive at such convictions as he has given us tonight and I think he deserves a great deal of credit and our cordial support.

Dr G. P. Rogers: This to me is one of the most important subjects in practical dentistry. The subject of tooth powder and tooth brushes is one that should receive our closest attention. I think if we recommend the tooth-washes and tooth powders on the market that have a large reputation, just because they are largely advertised, we are not doing what is right. We owe our patients the very best services in every line we can render, and our patients should be given to understand which tooth powder and which tooth-wash is best for the individual case, for obviously they cannot know unless they are advised. If we tell them to use a certain powder, because it is readily available and widely known without knowing what the constituents of such dentifrice may be, we are doing our patients great injustice, and belittling our professional intelligence. I think it is best to have a tooth powder that we know is not injurious and can be modified to suit individual cases. Of course those practicing oral prophylaxis can keep their patients under observation, and if they need a medicinal powder they can prepare it, or see that they get such a powder, and if they do not need that sort of a dentifrice they can recommend one that will not be injurious. When patients understand that just because a powder leaves a pleasant taste in the mouth that this is no sign that it is good for them. If they understand why they use the powder, and get one adapted to their needs, they will accomplish more with the powder than if they do not know.

DR. G. C. BOWLES: I want to say that I am glad to have heard Dr. Cook present this subject tonight and to know that he has somewhat changed his methods of treating teeth. The last time he spoke here on this subject his prophylaxis remedies were a stick and a rag; and he claimed great things were done by some people. I ran against a friend this afternoon who asked me what the subject was for this evening, and when I told him, he remarked that he suspected that a majority of the dentists knew practically nothing of the essential requirements of dentifrices to meet certain conditions that we meet within the mouth. We prescribe one powder that has been mentioned for every patient, and for all times, circumstances and conditions. I know for my own part that I was between the devil and the deep sea on this tooth powder proposition; until I heard Dr. Smith, I thought I knew something about tooth powders, I used the commercial samples that were sent to me. When Dr. Smith came along and preached cleanliness, I began to tell all patients that they should use more powder and polish their teeth for some minutes. After a while Professor Miller's articles appeared in the *Cosmos*, and I noticed that some of my patients had at that time what I had called erosion. I examine the teeth carefully now for tooth-brush erosions and I find the enamel in many cases cut pretty nearly through and deep grooves are cut, in many cases along the gingival border. Dr. Miller has pointed out this danger after many experiments, and proven conclusively that abrasion is many times caused altogether by tooth powders and brushing. Dr. Hoff says that chalk is not sufficiently abrasive. Dr. Miller found that the best English chalk on the market, as it is widely used in Europe would scratch glass, and if it would scratch glass it would scratch enamels, and by chalk alone he was able to cut into the pulp chamber.

After reading those articles I found that I did not know as much as I thought I did, and began to consider the tooth-powder proposition. It has been said that we should prescribe tooth powder, and I believe that we should. Some mouths come to us where the secretions in the mouth are normal and the teeth are well polished, to prescribe a tooth powder of a gritty nature for a patient of that kind is wrong. In some mouths the deposits are so thick on the teeth that you have to give the teeth a hard polishing before you reach the enamel. We have got to know the needs of the individual mouth and prescribe to meet these needs. I was talking some time ago to a manufacturer of a tooth powder; this manufacturer has sought for a tooth powder advocated and prescribed by dentists. He has gone through numbers of formulas appearing in the dental journals, and is doing what the average dentist never does, that is to give these powders scientific tests so that he knows what their chemical influences are, and what will be the outcome of the continued use of these agents, and this manufacturer tells me that it is the hardest thing in the world to get a tooth powder that is entirely satisfactory, a powder that will not cut glass, that is pretty nearly calcium carbonate. If we go to the drug store, or write out our prescription for our patients and send it to the drug store, it may be that they are not careful to secure clean and fine precipitated chalk, and so, we are not safe in prescribing for our patients through the druggist.

Dr. Cook said that the time required for antiseptics to destroy bacteria in the mouth was so great that the usual dentifrice was practically worthless. That statement has appeared in our literature time and time again and I suspect it must be true. However, recently there has appeared on the market a proprietary preparation called Alphozone and in a brochure published by the manufacturers they say that they have a remedy that will destroy, within one minute all bacteria in the mouth, in a solution strength of 1-1000. Dr. Ward and I were so interested in this matter that we visited the laboratory where this substance is made and we were assured that they have made careful and extended tests in this country and in Europe, with this preparation, and that it does destroy every bacterium in any mouth in one minute, and that so far no harmful effects of any kind have resulted to the mucous membrane in the mouth, nor can they see how any harm can follow the use of that preparation. If this is a fact, it may be of some use, although bacteria in the mouth is not all that we are seeking for after all.

I think sometimes we knock the commercial men unnecessarily. If any dentist ever devises a dentifrice that is ideal there will be a hundred commercial men that after preparation right away, who will put it up better than any dentist will put it up. Most of them, I suspect, are only too glad to get a formula that will fill the bill for all the requirements, and besides, we have got to be taught ourselves until we know the nature of the ingredients that are used in our dentifrices and until then we ought not to kick too vigorously because some one else steps in and does that which we ourselves have not attempted to do.

I have been using a tooth powder formula that I would like to have criticised, because I do not want to use it if it is not fit to use. It is:

| | | |
|---|---|---|
| R | Precipitated Chalk | 4 parts |
| | Magneseum carbonate | 3 " |
| | Powdered Orris Root | 2 " |
| | Bicarbonate of Soda | 1 " |
| | Oil of Wintergreen to flavor. | |

If it is too complex or has any injurious ingredients, I want to know it.

DR. W. A. DUMAS: I believe that I have the best dentifrice on the market, not because it is mine, but I think it is the best because it is a formula given me by one of the brightest and most scientific men that ever lived—Prof. W. D. Miller. I have had this formula now for some eight years and have watched its effect, and I know what it will do,

When Prof. Cook says that a tooth powder will only serve to cleanse the labial surfaces of teeth I must disagree with him, for the reason that I use in this formula materials which, when you place it in the mouth, will dissolve in the saliva and still be effective. I have taken a tooth with a large cavity from the mouth and placed it in on open dish and left it there forty days and it was unchanged. This preparation you can put in your mouth and it will not coagulate the albumen, as will some of the preparations on the market which containing 68% alcohol and with which you will find the drug stores filled. I would not put this article on the market if I did not think I had an article of value. I have used this for years, and under its use I have seen gums become as healthy as they can be. I do not claim, however, that the gums have become healthy from the use of this unless all calcarious deposits have been removed. If you remove the cause of pyorrhea or interstitial gingivitis you will find that this dentifrice that I have mentioned will do the work satisfactorily. The County Chemist, Dr. J. E. Clark, in a report, says that, after making "qualitative and quantitative analyses, I find that the powder contains materials that will act as antiseptic, alkaline, astringent and detergent, and contains nothing dangerous or injurious, but acts beneficially by hardening the gums and deodorizing the mouth and teeth." The formula contains Precipitated Chalk, Potassium Chlorate, Borax, Eucalyptus and other extracts.

Dr. G. F. Burke: I have been particularly interested in this paper, and I expected to hear a great many of the preparations that are on the market condemned, but before going away this evening, I should like to know what we are going to prescribe. I would like to hear Dr. Cook's correct formula, and I would like to know how we are to provide it for our patients. Shall we have our druggist put it up or shall we sell it in our offices. Dr. Cook spoke in his paper of a number of injurious ingredients that are found in the different preparations that are on the market, and I would like to know what they are. I should also like to ask Dr. Cook if salts of magnesia are commonly found in the preparations that are best known.

Dr. M. L. Ward: I expected Dr. Cook would condemn a great many commercial dentifrices and advocate some desirable ones, and along with his talk would diagnose some cases and prescribe suitable preparations for certain cases. In this respect it seems that he has left us entirely at sea. He has condemned pretty nearly all there is on the market, and has not given us much to take their places. One thing I would like to ask him in connection with the preparations that are put out with the claim that they prevent decay, to what extent he thinks the alkalies may be used in the mouth, to neutralize lactic acid, which produces decay, and to what extent such use will threaten the soft tissues in the mouth. Is bicarbonate of soda strong enough, in the preparations commonly put out, to neutralize lactic acid which produces decay, and at the same time not injure the soft tissues in the mouth.

Dr. T. R. Buttrick: I believe that a powder is absolutely necessary in tooth cleanliness. Dr. Cook has given us many reasons why it should not be used, but I do not believe any mouth can be kept clean with a wash and brush only. I think you have got to have an abrasive. We eat without proper mastication, the saliva does not flow freely, and it is very difficult to keep the teeth clean, and if they are neglected for any length of time they begin to decay. I have been in the habit of urging my patients to use tooth powder, and use it generously twice a day with a proper brush as I think this necessary to keep the bacterial deposits off the teeth. This may cause some abrasion. I have read the articles of Dr. Miller but I am not convinced that we should not use powders. I have seen mouths where it was very evident erosion had occurred, and there was also very evident lack of tooth brushing. I have one mouth under my care in which one incisor has a very perceptible abrasion and not another tooth in the mouth is touched.

I can't believe that that one tooth is thus attacked by brushing the whole set; there must be some chemical action responsible besides the tooth brush and powder.

My own idea of a tooth powder is largely calcium carbonate with some magnesia, and I would also add a little powdered cuttlefish for the abrasive. A small percentage of potassium chloride is a good antiseptic. For acid conditions we want something alkaline bicarbonate of soda. A little sweetening and flavoring and a little powdered soap for detergent and lubrication are also valuables. I believe you will do less harm with such a tooth powder than will result if you leave the teeth unclean and in a condition to invite bacterial development.

Dr. Bowles: I wish every one would read Dr. Miller's articles in the January, February and March "Dental Cosmos." Dr. Miller comes pretty nearly being an authority and what he has to say on this subject is the result of a careful research and should be read and practiced by every dentist in the land. So many of us have opinions but they are based on nothing but clinical observation. Let us study the works of such a man as Miller before we have convictions.

Dr. Don Graham: While I have not always agreed with Dr. Cook, and do not now, yet I value his services to us on this important topic. Most of us have not the time or ability for studying these subjects as he has. I do not understand that Dr. Cook has a large family dependent on him or anything like that, and that he has independent means or perhaps he could not do this work. What he has given us tonight is the most valuable contribution we have had on this subject in this society for many years, and that we shall all profit by it there is no doubt. I am some interested in oral prophylaxis, and am trying to follow out the suggestions and teachings of our worthy friend Dr. D. D. Smith. I have prescribed powders for particular patients, and have had druggists put them up, but I am not going to tell you the formulas. Since I began practicing oral prophylaxis, to the best of my ability I have come to the conclusion that I do not care very much whether my patients use powder or liquid dentifrice; but I shall experiment with Dr. Cook's formula, as it seems to have rational qualities. Any one following oral prophylaxis methods can keep his patients mouths so clean that a very little brushing will suffice. So far as this abrasion is concerned, I have always believed that most of the erosions, or abrasions of the teeth come from some chemical action on the tooth substance, rather than from any mechanical reagent. Common sense ought to teach us that all harsh powders will wear the teeth: however, I have a patient whose mouth is kept in almost as good condition as any mouth I have ever seen, and she brushes her teeth with hand sapolio. My brother came to me after he got through college and I found that he had never brushed his teeth at all and he had but one decayed tooth in his mouth. I filled that cavity and taught him how to brush his teeth and he has not had a cavity in his teeth since. But such a case does not prove that he does not need to have a proper powder prescribed for his use.

Dr. Spalding has suggested a thought that is most important that the rotary motion be given the brush, which does away with the injury to the gums and the necks of the teeth. I think it is very important that we teach our patients how to rotate the brush, not up and down, nor horizontally, but rotate it all the time, and this will remove, or at least dislodge the adhering particles better than any other method. I have a patient with whom I was talking this evening; when he came to me more than six months ago, his mouth was just reeking with suppuration from pyorrhea; the pus was oozing out all around and he was sick, and about to quit business he was so despondent. His physician told him that that pus oozing out of the gums would make him sick. His dentist had told him that he would have to lose his teeth in a short time; but he could help him some. He had him come back regularly every month and he painted his gums with iodine and told him to use glycothymoline as a mouth-wash. When he came to me the gums were in a fairly healthy looking condition, but the pus was oozing out around nearly every tooth. Every one of those teeth was covered with deposits from the gum margins to the end of the roots all the way around. I speak of this case to emphasize the importance of scaling the roots thoroughly under the gum.

I had a patient come to me this week who had been treated by a good dentist for pyorrhea within a month and discharged cured but the gum was standing away from all of the teeth, and deposits were upon every one of them. That dentist did not know what oral prophylaxis means.

DR. COOK: I have awfully cold feet. I knew this paper of mine would not be satisfactory to you or at least for some of you—and that is just the way with tooth powders, there are only a few that will satisfy. It is not the usual thing for a scientific investigation to coincide constantly with clinical experience. That has seldom been so, with the exception of a few instances. In the treatment of the teeth and the oral cavity, we observe things that we cannot explain, nor can any one else explain satisfactorily to us or to themselves: but we shall have to keep chewing away at these powders until we find out whether Miller's theory or his experiences were correct or incorrect. This is the only way that we can ascertain whether they are or not. We have got to duplicate several times what he has done, before we can say that we do not believe in what he did. I have used tooth powders ever since I began practice and I have not got an erosion in my mouth; and there are hundreds of patients where you may see an erosion appear in some particular place or on a particular tooth, and where the tooth brush never touched it. We have all observed this. I have not said and do not believe, that the tooth brush and tooth powders were the true and only cause of erosion of the teeth; be it far from me to think that. But a tooth powder may cause irritation of the mucous membrane, and as a result of continued use a mucoid degeneration of the tissue of the mouth follows, which may produce a degeneration in the mucus that is secreted from the mucus glands, and when this is abnormal, in most instances it is acid, and that is just what is produced by many of the tooth powders and tooth washes in use. There is no question in my mind about the value of Miller's experiments, for I have duplicated some of the experiments to my own satisfaction and found his experiments were absolutely correct. I can take the polish off of glass with any tooth powder that is on the market today, and I cannot make one myself that I cannot do it with; that is the reason that I do not recommend a tooth powder to you. If you brush the teeth with a tooth powder for 25 years, twice each day for five minutes, it will make a great impression on some teeth. I have produced a fine erosion in a patients mouth with an orangewood stick and water. It is not a question in my mind whether we should or should not use tooth powder, I know we should; but I say that there is not a formula and there never can be, that you can prescribe for any mouth under any and all circumstances. You may take this dentifrice of Dr. Dumas,—and doubtless it is just as good as any of the rest, and perhaps a little better, as long as Dr. Miller has recommended it, and it is his formula, we should take it as being worthy of consideration and you can take the polish off a piece of looking glass with it. But Dr. Miller in all his 25 years work, never recommended to the profession any particular mouth-wash or tooth powder that you could use universally. There are certain mouth-washes and certain tooth powders that we may prescribe ,but the question in our minds now is, shall we prescribe a tooth powder, or mouth-wash, or paste, for a perfectly healthy mouth and sound teeth, to be used three times a day, as long as the patient wishes to scrub his teeth? People are being educated to the fact that they must use these powders plentifully. This is a wrong education. Because you drop a little eye water in the eye when it is sore it does not follow that you should keep dropping it in there after the eye gets well; if you did you would produce degeneration of the cornea, and the result would be blindness. The question that is interesting to me, most of all, is the fact that we are drifting too much in thinking that the bacteria that are in the mouth are pernicious at all times. They may be important, and we should treat them accordingly to the laws that govern all physiological phenomena. We should not treat the mouth any more zealously than we should treat our hands or feet. Do you medicate your hands and feet every night with abrasive and antiseptic lotions? If they get sore we treat them, and that is what we should do with the mouth and teeth. The ordinary washes I think will suffice in the ordinary cases of healthy mouths.

Dr. Hoff recommends an abrasive tooth powder. That would be a proper thing if you had a mouth which required the oral prophylaxis treatment. If you have a mouth in which you have mucoid deposits around the necks of the teeth, and perhaps other deposits under the gingival border of the gum, or have a roughening of the surface of the tooth, then you have got to use abrasives, but you have not got to use them all the time. Change your formula as you go along. You can make proper powders, or have them put up in your offices. You can get a boy for 25 cents a day, who can take the mortar and grind the powder as fine as you can get it or so that you can sift it through two thicknesses of ordinary silk.

Dr. Smith, of Philadelphia, has brought to the minds of the profession one essential thing, that of seeing your patients, who need to be seen, very often. Heretofore we had been in the habit of seeing our patients as may be convenient and filling their teeth, and telling them to come back in a year, or perhaps only after they have passed through a stage of typhoid fever, or childbirth, or something of that kind, and when they do come back, probably their teeth will have become decayed, and in the meantime they have probably developed a lot more oral conditions that need attention. We have educated people badly by telling them to go to the apothecary around the corner and get some of the "sopa suds" that somebody is making, and to rub the teeth white. We have got to get out of this habit, just the same as we must quit "throwing in" the cleaning process when we fill a tooth for a dollar. Some of our best men are practicing that kind of dentistry. We should get to the point of saying to our patients, "here is a condition that needs attention; it is not a filling, but must be treated or eventually you are going to lose your teeth." We must get our patients to think about the conditions which we have got to treat them for and that their teeth are all-important. A lady told me not long ago that when she went into a dentist's office, the dentist told her "you are going to lose your teeth," and then he said "but it is all right, you have got a good roof to hold artificial teeth in." "Well," I said to her, "I suppose if you went into a surgeon's office with a sore toe, he would say, "well, you can use the stump, we will just cut this off."

I wish I did have a formula to give the profession, I would just bundle it up and send it to you, and if you wanted me to manufacture it, why I would manufacture it honestly, if I thought it would do any good. In looking through my office and laboratory the other day, I counted nearly a hundred different formulas of powders and mouth-washes that I could prescribe. One of them might be good for one case and another for another. Oftentimes I have prescribed these so indiscriminately that they have produced bad results where I expected good results, but this is characteristic of the most scientific therapeutics.

The thing we are looking for, of course, is the betterment of our patient. When you have a case, don't think that you are going to diagnose it and absolutely establish a treatment for that particular case, mouth-wash, tooth powder or what not the first time you see the patient. That I do not think anybody will do with any degree of intelligence.

You can kill bacteria in the mouth easier with a normal salt solution than with anything else, but if you are going to prescribe a mouth-wash or tooth powder, don't think that you are going to get it so simple as to give it in one ingredient. You will produce better effects if you put in several salt solutions, but don't get the quantity too great to be practically soluble by the water in the mouth.

There are some cases where I would just as soon use some of these proprietary remedies as I would anything else, but I would not prescribe them universally. If we could ever get to a point where we had a committee from the various state societies, or the national society to take up and investigate such matters, and publish the investigations to the world, just exactly what they found, as to how these remedies are prepared this would help some as there are many of these remedies that are better prepared than the average druggist would put them up for us. If you could train your druggist as to what you wanted, or at your leisure hour went in and did it yourself, you would learn a lot of things about this business and get the knowledge you need. The therapeutics of diseases of the mouth should be just as scientific as the therapeutic treatment of malarial fever; we have not so many varied conditions in the mouth but what they can be treated successfully, especially if we use preventive treatment and arrest certain disease processes that have been started. The point that I wish to bring out tonight is this; Don't let the Commercial man do all the thinking for you. Think more about the case that you have in hand. This is a question of therapeutics, pure and simple, and, if you have the knowledge that you should have, there is no one that can settle for you as well as you can the proper treatment of each case. I could not go into your office and take your cases and treat them as well as you can, until I had got some line on the conditions that were present and the peculiarities of your patients.

You cannot make one treatment universal to everything, and you must use your own judgement about it to a large extent. That is just the point that I want to emphasize, and that is the reason I read this paper just as I did, to emphasize that one point—that you cannot prescribe any one tooth powder or tooth wash that is going to answer for all cases at all times and in all circumstances.

